# Odile Bain (April 28, 1939–October 16, 2012): A Life Dedicated to Systematics and Biology of Filariae

**DOI:** 10.1371/journal.pntd.0002565

**Published:** 2014-02-13

**Authors:** Coralie Martin

**Affiliations:** UMR 7245 MCAM MNHN CNRS, Muséum National d'Histoire Naturelle, Paris, France; Lindsley F. Kimball Research Institute, New York Blood Center, United States of America

[Fig pntd-0002565-g001]Odile Bain was born on April 28, 1939, in Dalat, Vietnam, where her father, a military officer, was based. She went to high school in Dakar, Senegal, and graduated in biology in Rennes (France) in 1960. She taught biology at a high school for two years and then worked as a teaching assistant at the Faculty of Rennes in 1962. In 1963, she started a postgraduate course in histology in Paris. Odile moved to the Natural History Museum in Paris, France, in September 1964 to join the Helminth Zoology laboratory of Prof. Alain Chabaud. It was there that she began to build an incredible career in parasitology. She first graduated with her PhD (“thèse de 3ème cycle”) in histology in April 1968. Then, in April 1971, she was awarded her “Doctorat d'Etat es Sciences Naturelles” for her work on “Evolution des filaires chez le vecteur: morphologie larvaire et mécanismes du passage des microfilaires dans l'hémocèle.” In the meantime, she was appointed by the CNRS (*Centre National de la Recherche Scientifique*) in October 1965 as an undergraduate research assistant; then she was granted a position as a CNRS research associate (*chargée de recherche*) in October 1971 and, later, in January 1984, as a CNRS research supervisor (*directeur de recherche*).

**Image 1 pntd-0002565-g001:**
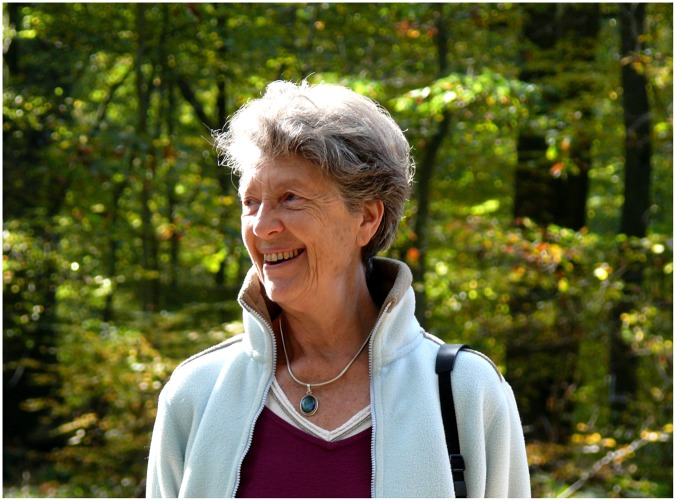
Photo courtesy of Kerstin Junker (Ile de France, 2008).

The laboratory is a parasitology history lesson in itself. It was founded in 1961 by Prof. Chabaud and gathered specialists of different groups of “worms” and protozoa. Prof. Chabaud supervised several teams of research on parasitic nematodes, trematodes, cestodes, and protozoa. The laboratory was originally located on rue Cuvier but moved to its present location on rue Buffon in 1981. The laboratory quickly established itself as a centre of excellence, but it retained an unbridled hospitality supported by good food and a sucession of resident canine friends.

It is in this laboratory that Odile honed her parasitological and morphological skills. She had a passion for biology and microscopy that was unrivalled and universally recognised. In her 50-year career, she published more than 360 articles (Text S1) and had 30 years of successive contracts with the World Health Organization (WHO), the Edna McConnell Clark Foundation, and the European Union. In 1974, she received the bronze medal of the CNRS, and in 1984, she won the Foulon prize from the French Academy of Sciences for her work on the zoology and parasitology of filarial infections. She was also the curator of the zooparasitic nematodes collection at the French National Natural History Museum, Paris.

Odile's primary expertise was the systematics of filariae, and her intimate knowledge of the biology of filariae led to characterization of the phyletic relationships between filarial lineages and those of non-filarial nematodes. Odile's group studied a large number of filarial species collected in many countries, which led to countless worldwide collaborations. Over time, the scope of her research expanded to encompass the medically relevant filarial species that cause diseases of humans in the tropics. In these studies she investigated aspects of vector biology that relate to filarial worm infection with the end goal of devising a means to control transmission. She also developed a strong interest in finding and identifying the vectors of lesser-known zoonotic filarial species. Her knowledge of animal filariae also resulted in the establishment of experimental models of filariasis in rodents (*Monanema martini* with skin-dwelling microfilariae, *Molinema dessetae*), including *Litomosoides sigmodontis*, which is the only filaria capable of establishing patent infections in laboratory mice. Use of these models to study host–parasite interactions has significantly advanced the study of filarial disease, chemotherapy, and immunology.

Besides being a most highly respected scientist who strove for only the highest quality research, Odile Bain was an eternal and energetic optimist who welcomed everyone with friendliness, and whose enthusiasm and curiosity were infectious. We all deeply feel her loss and will remember the smiling Odile, always ready to take on new challenges in science.

## An Overview of Odile Bain's Achievements

### 1. Filariae systematics

A large number of species from around the world were described or redescribed by Odile Bain. She published a dichotomous key of the Filarioidea, together with hypotheses on the evolution of the superfamily, including the position of the human filariae, using, initially, traditional morphological methods and, later, supported by the first molecular phylogeny of these parasites. She described new filarial species, and she largely studied genera and species of the Onchocercidae such as:

- The genus *Onchocerca*
- The *Dipetalonema* lineage
- The genus *Dipetalonema* sensu stricto parasite of South American monkeys
- The genus *Mansonella* and its sub-genera
- The genus *Molinema* from caviomorph rodents
- The genus *Cercopithifiaria* from mammals worldwide
- The genus *Litomosoides* from neotropical rodents, marsupials, and bats
- The genera *Litomosa* and *Josefilaria* from old-world bats
- The filariae *Edesonfilaria* from Dermaptera, *Chabfilaria* from Xenarthra, and *Cherylia* from South American marsupials
- Filariae of birds: *Dessetfilaria*, *Andersonfilaria*, and *Versternema*.
- Filariae of reptiles
- Filariae of amphibians
- The *Stephanofilaria*, skin parasites of ungulates
- *Brugia malayi*: geographical strains identified by discriminative characteristics based on cuticular ornamentation of the male posterior end

She also largely contributed to the study of filarial zoonoses, such as the ones due to *Meningonema perruzzi*, a parasite of cercopithecid monkey; *Mansonella rodhaini*, a parasite of anthropoid apes; *Onchocerca dewittei japonica*, a parasite of wild boars in Japan; and more recently, *Onchocerca lupi*, a parasite of dogs.

In the last decade, she was studying the relationship between *Wolbachia* and filariae and their coevolution.

She also worked on other orders of nematodes, including:

- Muspiceoidea, with the reinterpretation of the anatomy of *Riouxgolvania* and *Dioctowittus*; new descriptions of the genera *Lukonema*, *Pennisia*, and *Lappnema*; and muspiceoid infective larva in *Simulium*; a dichotomous key has also been published.
- Trichinelloidea (*Capillaria* sensu lato, *Trichomosoides nasalis*)
- Rhabdiasidae and other parasites of terrestrial cold-blooded vertebrates (*Chabirenia*, *Rhabdias*)
- Oxyuroidea and Oxyuridae, with a focus on traumatic insemination, the organ of De Mann, and *Gyrinicola* from tadpoles

### 2. Vectors and transmission of filariae

One of her earliest interests was filarial morphogenesis in the vector. She clarified the fate of the microfilarial cells called R1, R2, R3, and R4 and discovered that R2, R3, and R4 cells form the rectal glands and do not divide. The R1 is a primary l mesenchymal cell that divides and produces muscle cells. The morphology and development of microfilariae and their organs were determined through comparative studies of filariae from amphibians, reptiles, birds, and mammals.

She also focused on identification of infective larvae from vectors, a difficult task, but one that was essential for estimating transmission intensity in endemic areas. At the request of WHO, she published an atlas of infective stages of filariae, using simple characteristics as a mean of identification that can be used in the field.

Her third interest was the regulation of infection in the vector, after ingestion of microfilariae or during development.

Odile established that in many filaria–vector relationships, the number of infective larvae produced is not proportional to the number of microfilariae ingested. She described two opposing types of regulation processes: limitation versus facilitation. Limitation means that the proportion of microfilariae arriving in the haemocoel decreases when the number of microfilariae ingested increases. Such examples are numerous and include *Onchocerca volvulus* in *Simulium sirbanum* and *S. damnosum* in the African savannah; filariae of lizards, marsupials, and South American rodents in various strains of *Aedes aegypti*; and *Wuchereria bancrofti* in *A. polynesiensis*. Facilitation indicates that the proportion of microfilariae arriving in the hemocoel increases with the number of microfilariae ingested, as for *W. bancrofti* in *Anopheles gambiae* A in West Africa. Both types of regulation are due to changes in the vector's stomach wall in response to stimulation by the microfilariae, causing an increased secretion of the peritrophic membrane (*S. damnosum*, *S. sirbanum*), and either death (*A. aegypti*) or hypertrophy of the digestive cells affected by microfilariae. These reactions suggest a comprehensive immune response in the stomach wall. Ivermectin administered to the host modifies these vector–filariae relationships. Other patterns of regulations were observed during the ingestion of microfilariae, particularly following ivermectin treatment for onchocerciasis.

Odile also demonstrated different interactions within the adipose tissue where the parasites develop in mosquitoes (*Molinema dessetae* and *Breinlia booliati*). For the first species, blood meals activate both the infected syncytium and healthy adipocytes (vitellogenesis cycle is unchanged). In *B. booliati*, the infected syncytium is unresponsive to the blood meal stimulus. The yield of *M. dessetae* infective larvae is better when mosquitoes continue to take blood meals after ingestion of microfilariae.

### 3. Models of filariasis

Filariae infect all vertebrate phyla except fishes, and many hundreds of species from 94 currently known onchocercid genera have been described. Most species exhibit a marked host specificity, and this includes the important medical filariae such as *Onchocerca volvulus*, the causative agent of river blindness, and *Wuchereria bancrofti*, one of the filariae responsible for lymphatic filariasis or elephantiasis. This situation prompted the search for animal models that mimic the various biological and developmental characteristics of human parasites and could provide a screening system for drugs and, eventually, experimental vaccines.

Odile's research led to the discovery of:

- Diurnal periodicity of microfilariae of *Molinema dessetae* in the blood of *Proechimys oris,* the para spiny rat from Brazil.
- Description of dermal lesions associated with skin-dwelling microfilariae of *Monanema martini* in *Lemniscomys striatus* and *Arvicanthis niloticus, Cercopithifilaria roussilhoni* from *Atherurus africanus* (the African bush-tailed porcupine), and *C. johnstoni* from Australian rodents and marsupials. These models have facilitated the study of filarial pathogenesis and complemented work with bovine *Onchocerca* species. Both rodent and bovid modeles have been used in drug trials.
- The observation that the L3 to L4 moult of *Onchocerca* and *Dirofilaria* filariae occurs just two days after infection of the vertebrate host, contrasting with the later moulting seen at seven days post infection in most other filariae, including *Brugia, Wuchereria, Litomosoides, Acanthocheilonema, Monanema*, and *Loa*. These biological events, as well as genome sequence data and the typology of endosymbiont *Wolbachia*, now question the validity of subfamily status of Onchocercinae and Dirofilariinae.
- Detailed description of biological features to analyse filarial infection, such as kinetics of the recovery of filariae in relation to the number of infective larvae inoculated in rodents, the relationship of filariae with the lymphatic system and cardiopulmonary circulation, the blood-feeding behaviour by filariae in the host, etc.

### 4. The *Litomosoides sigmodontis* murine model

Odile's greatest contribution to the development of filarial models was the demonstration that *Litomosoides sigmodontis*, a natural parasite of South American cretids, can produce patent infections in laboratory BALB/c mice.

*L. sigmodontis* started its “scientific career” in 1945 as *L. carinii*. Earlier, a filarial parasite was discovered and described by Chandler in 1931 in a Muridae Sigmodontinae, the cotton rat, *Sigmodon hispidus*, captured in Houston, Texas. Chandler used this material to describe the type species of a new genus, *Litomosoides*, in which was noted a uniquely long buccal capsule. This author transferred to this new genus a filaria described by Travassos (1919), *Filaria carinii*, that had been recovered from an undetermined Sciuridae, *Sciurus* sp. (*Phyrhonotus*?), São Paulo, Brazil. Shortly after, Vaz synonymized all the species of *Litomosoides*, including *L. carinii* and *L. sigmodontis*, into a single species referred to as *L. carinii*.

However, in the late 80's, Odile studied anew the nomenclatural type specimens of *L. carinii* from squirrels and *L. sigmodontis* from cotton rats and confirmed their distinctive morphological characters. As a consequence, the filaria now being maintained in laboratory mice has reverted to its original name of *L. sigmodontis* Chandler, 1931.

The genus *Litomosoides* is very diverse, with 33 species distributed in the New World. A closely related genus, *Litomosa* York and Maplestone, 1926, is largely restricted to the Old World. Both genera are parasitic in bats, but *Litomosoides'* host range also includes rodents and some marsupials. *Litomosoides* species from rodents and marsupials are morphologically very similar, indicating a recent diversification of *Litomosoides* in these zoologically distant hosts. Odile suggested that *Litomosoides*, like *Litomosa*, are primarily parasites of bats. Chiropterans are ancient (fossils were found as early as the Eocene), and they are present on the South American continent. This continent was isolated during much of the Tertiary, but during the Pleistocene era, the junction of the two Americas was achieved. Holarctic fauna, e.g. rodents, migrated to the south, where they diversified. This event promoted the capture by these newcomers of paleo-endemic *Litomosoides* from bats. Parasitism of marsupials by *Litomosoides* seems contemporary, and not prior to this second wave of diversification, because only the smaller species are parasitized.

*L. sigmodontis* is now established as a model of filarial infections in mice, where it presents with a spectrum of parasitological and immunological presentations that mimic those seen in human infections. BALB/c mice are susceptible to infection and can produce patent infections, although some BALB/c can be amicrofilaraemic. Other mouse strains, such as C57BL/6, are resistant to infection, and thus when Odile and her colleagues published these observations in 1992, the research community was finally provided with a tractable system for investigation of the immunology of filarial infections.

The following 20 years saw publications of detailed descriptions of the development, maturation, and behaviour of *L. sigmodontis* in mice, while immunological studies have defined immune responses evoked by filarial infections and the regulatory processes that lead to expression of protective immunity. Proof-of-principle of immunisation against filarial parasites has been obtained following vaccination with irradiated attenuated L3 larvae, recombinant protein, and recombinant DNA.

These achievements are all owed to Odile's ability to integrate classical zoology with high technology. Today, her generosity and enthusiasm continue to drive her colleagues along the road to elimination of filarial infections as public health problems, while maintaining a fascination and curiosity for a unique group of animals.

## Supporting Information

Text S1 List of Odile Bain's publications.
(DOC)

