# Supplementary material for: Odile Bain (April 28, 1939–October 16, 2012): A Life Dedicated to Systematics and Biology of Filariae
Source: PLoS Negl Trop Dis. 2014 Feb 13;8(2):e2565. doi: 10.1371/journal.pntd.0002565 (PMC3923714; doi:10.1371/journal.pntd.0002565)
Supplement: Text S1 — List of Odile Bain's publications. (DOC) [file pntd.0002565.s001.doc]

Supporting Information S1

Dr Odile Bain’s publications

1. Rioux JA, Coluzzi M, Houin R, Bain O, Baudouy JP (1964) Présence de *Phlebotomus ariasi* Tonnoir, 1921 en Italie du Nord. Bull So Path Exot 57: 966-971.
2. Chabaud AG, Bain O (1965. Spirurides parasites d’Oiseaux malgaches: 3ème note. Bull Mus Natl Hist Nat 2 sér 37: 173-185.
3. Chabaud AG, Golvan Y, Bain O, Brygoo E (1965) *Gynopoecilia pseudovipara* n. gen. n. sp, et cycles endogènes chez les Nématodes zooparasites. CR Acad Sci sér D 260: 4602-4604.
4. Chabaud AG, Bain O (1965) *Aelurostrongylus pottoi* n. sp, Metastrongylide parasite de Primates et remarques sur les affinités entre les Nématodes de Carnivores, d’Insectivores et de Lemuriens. Ann Parasit Hum Comp 40: 569-573.
5. Chabaud AG, Golvan Y, Bain O, Brygoo E (1965) Etudes systématiques et données biologiques sur un Nématode Rhabditoïde parasite de Mantes à Madagascar. Bull Soc Zool France 40: 231-241.
6. Bain O (1965) Oxyures parasites de la Courtillière *Gryllotalpa africana*, à Madagascar. Ann Parasit Hum Comp 40: 659-676.
7. Chabaud AG, Bain O (1966) Description de *Hartwichia rousseloti* n. gen, n. sp, Ascaride parasite de Crocodile et remarques sur la famille des Heterocheilidae Railliet et Henry, 1912. Bull Mus Natl Hist Nat 2 sér 37: 848-853.
8. Bain O (1966) Particularités biologiques de la Filaire *Dipetalonema viteae* chez l’hôte intermédiaire. CR Acad Sci sér D 263: 658-659.
9. Bain O (1967) Biologie larvaire et mécanismes de transmission de la filaire *Dipetalonema viteae*. Ann Parasit Hum Comp 42: 211-267.
10. Chabaud AG, Bain O, Houin R (1967) Nématodes du Potamochère malgache. Ann Parasit Hum Comp 41: 599-606.
11. Chabaud AG, Bain O, Puylaert F (1966) Description de 3 nouveaux Nématodes Molineinae et considérations sur le caractère archaïque de cette sous-famille. Bull Mus Natl Hist Nat 2 sér 38: 904-920.
12. Chabaud AG, Bain O, Tcheprakoff R (1967) Redescription de *Galeiceps cucullus* (Linstow, 1899) et remarques sur l’osmo-régulation des Nématodes Anisakides. Ann Parasit Hum Comp 42: 321-326.
13. Petter A, Bain O, Orcel L (1967) *Larva migrans* expérimentale chez un Primate, provoquée par un Ascaride de Serpent. Ann Parasit Hum Comp 42: 207-210.
14. Rioux JA, Golvan Y, Houin R, Juminer F, Bain O. (1967). Contribution à l’étude écologique des Leishmanioses dans le sud de la France. Ann Parasit Hum Comp 42: 561-604.
15. Bain O (1967) Diversité et étroite spécificité parasitaire des Filaires de Chauves-Souris confondues sous le nom de *Litomosa filaria* (Van Beneden, 1872). Bull Mus Natl Hist Nat 2 sér 38: 928-939.
16. Bain O, Ghadirian E (1967) Description d’une nouvelle espèce de *Dioctowittus* (Nématode) et note sur la position systématique du genre. Ann Parasit Hum Comp 42: 643-650.
17. Bain O, Chabaud AG (1968) Description de *Riouxgolvania rhinolophi* n.g, n. sp, Nématode parasite de Rhinolophe, montrant les affinités entre Muspiceoidea et Mermithoidea. Ann Parasit Hum Comp 43: 45-50.
18. Bain O (1969) Morphologie des stades larvaires d’*Onchocerca volvulus* chez *Simulium damnosum* et redescription de la microfilaire. Ann Parasit Hum Comp 44: 69-82.
19. Bain O, Hocquet P (1968) *Ackertia dorsti* n. sp, parasite de la Viscache Lagidium peruanum. Bull Mus Natl Hist Nat 2 sér 40: 399-402.
20. Bain O (1968) Remarques au sujet d’une nouvelle Filaire de Caméléon malgache, proche de *Foleyella brevicauda*. Bull Mus Natl Hist Nat 2 sér 40: 802-806.
21. Bain O, Brunhes J (1968) Un nouveau genre de Filaire, parasite de Grenouilles malgaches. Bull Mus Natl Hist Nat 2 sér 40: 797-801.
22. Chabaud AG, Durette-Desset MC, Bain O, Leger N, Albaret JL (1969) *Potamopyrgus* et *Bullins* en Corse (Août 1969). Ann Parasit Hum Comp 44: 821-824.
23. Bain O (1969) Etude morphologique du développement larvaire de *Foleyella furcata* chez *Anopheles stephensi*. Ann Parasit Hum Comp 44: 165-172.
24. Bain O, Philippon B (1969) Recherche sur des larves de Nématodes Secernentea trouvées chez *Simulium damnosum*. Ann Parasit Hum Comp 44: 147-156.
25. Bain O (1969) Cycle d’une Filaire d’Agame, *Saurositus agamae hamoni* n. ssp. (Eufilariinae) chez *Anopheles stephensi*. CR Acad Sci sér D 268: 2439-2441.
26. Bain O (1969) Développement larvaire de *Saurositus agamae hamoni* n. ssp, Eufilariinae parasite d’Agame en Haute-Volta, chez *Anopheles stephensi*. Ann Parasit Hum Comp 44: 581-594.
27. Bain O, Vassiliades G (1969) Cycle évolutif d’un Dicheilonematinae, *Serratospiculum tendo*, Filaire parasite d’un Faucon. Ann Parasit Hum Comp 44: 595-604.
28. Bain O, Philippon B (1969) Mécanisme du passage des microfilaires à travers la paroi stomacale du vecteur; son importance dans la transmission de l’onchocercose. CR Acad Sci sér D 269: 1081-1083.
29. Rioux JA, Chabaud AG, Bain O, Quentin JC, Durette-Desset MC (1969) Infestation spontanée de *Phlebotomus ariasi* par *Rictularia proni*, Spiruride parasite de Mulot. Les terriers de Mulots sont-ils des gîtes larvaires à Phlébotomes? Ann Parasit Hum Comp 44: 757-760.
30. Bain O (1970) Etude morphologique du développement larvaire de *Foleyella candezei* chez *Anopheles stephensi*. Ann Parasit Hum Comp 45: 21-30.
31. Bain O (1970) La cellule R1 des microfilaires (Nematoda) initiale du mésenchyme. Ann Parasit Hum Comp 45: 227-235.
32. Bain O, Philippon B (1970) Mécanisme de la traversée de la paroi stomacale par les microfilaires chez *Anopheles stephensi* et *Simulium damnosum*. Mise en évidence d’un séjour des microfilaires dans l’épithélium digestif. Ann Parasit Hum Comp 45: 295-320.
33. Bain O (1970) Morphologie larvaire de *Setaria labiatopapillosa* (Nematoda, Filarioidea) chez *Aedes aegypti*. Ann Parasit Hum Comp 45: 431-439.
34. Chabaud AG, Puylaert F, Bain O, Petter AJ, Durette-Desset MC. (1970) Remarques sur l’homologie entre les papilles cloacales des Rhadbitides et les côtes dorsales des Strongylida. CR Acad Sci sér D 271: 1771-1774.
35. Bain O (1970) Cycle évolutif de l’Heterakidae *Stongyluris brevicaudata* (Nematoda). Mise en évidence de deux mues dans l’oeuf. Ann Parasit Hum Comp 45: 637-653.
36. Bain O (1971) Evolution des Filaires chez le vecteur: morphologie larvaire et mécanismes du passage des microfilaires dans l’hémocèle. Thèse Doct. Etat Sc. Nat, Fac Orsay, 3 Avril 1971,134 pp.
37. Bain O (1971) Transmission des Filarioses. Limitation des passages des microfilaires ingérées vers l’hémocèle du vecteur; interprétation. Ann Parasit Hum Comp 46: 613-631.
38. Bain O (1972) Recherches sur la morphogenèse des Filaires chez l’hôte intermédiaire. Ann Parasit Hum Comp 47: 251-303.
39. Bain O, Brengues J (1972) Transmission de la Wuchérériose et de la Sétariose bovine: Etude histologique de la paroi stomacale d’*Anopheles gambiae* A et d’*Aedes aegypti* par les microfilaires. Ann Parasit Hum Comp 47: 399-412.
40. Prod’Hon J, Bain O (1972) Développement larvaire chez Anopheles stephensi d’Oswaldofilaria bacillaris, Filaire de Caïman sud-américain, et redescription des adultes. Ann Parasit Hum Comp 47: 745-758.
41. Brengues J, Bain O (1972) Passage des microfilaires de l’estomac vers l’hémocèle du vecteur, dans les couples *Wuchereria bancrofti*-*Anopheles gambiae* A, *W. bancrofti*-*Aedes aegypti* et *Setaria labiatopapillosa*-*A. aegypti*. Cah ORSTOM sér Ent Med Parasitol 10: 235-249.
42. Philippon B, Bain O (1972) Transmission de l’Onchocercose humaine en zone de savane d’Afrique Occidentale, passage des microfilaires d’*Onchocerca volvulus* Leuck. dans l’hémocèle de la femelle de *Simulium damnosum* Th. Cah ORSTOM sér Ent Méd Parasitol 10: 251-261.
43. Bain O, Durette-Desset MC (1973) Cycle de *Skrjabinofilaria skrjabini*, Filaire de Marsupial sud américain. Ann Parasit Hum Comp 48: 61-79.
44. Bain O, Vaucher C (1973) Développement larvaire de *Diplotriaena tridens* (Nematoda : Filarioidea) chez *Locusta migratoria*. Ann Parasit Hum Comp 48: 81-89.
45. Vaucher C, Bain O (1973) Développement larvaire de *Dracunculus doi* (Nematoda) parasite d’un serpent malgache et description de la femelle. Ann Parasit Hum Comp 48: 91-104.
46. Bain O (1973) Cycle d’une Filaire de Rongeur passant par Moustique. CR Acad Sci sér D 276: 2689-2690.
47. Bain O (1973) Une nouvelle Filaire de Rongeur sud-américain, *Dipetalonema dessetae* n. sp. (Nematoda, Filarioidea). Bull Mus Natl Hist Nat 3 sér Zool 116 (90): 309-316.
48. Bain O, Haesevoets E (1974) Affinités entre deux filaires de l’appareil circulatoire, l’une parasite de Bovidés, l’autre de Cervidés: *Cordophilus sagittus* (Linstow, 1907) et *C. abramovi* (Oshmarin et Belous, 1951), nov. comb. Ann Parasit Hum Comp 49: 119-122.
49. Bain O, Bussiéras J, Amégée E (1974) Dualité d’*Onchocerca volvulus* de l’homme et d’*Onchocerca* sp. Cameron, 1928, du Bétail. Nouvelles espèces d’Onchocerques bovines au Togo. CR Acad Sci sér D 278: 369-372.
50. Chabaud AG, Bain O (1974) Données nouvelles sur la biologie des Nématodes Muspicéides fournies par l’étude d’un parasite de Chiroptères: *Lukonema lukoschusi* n. gen. n. sp. Ann Parasit Hum Comp 48: 819-834.
51. Bain O, Beaucournu JC (1974) Larves infestantes de *Dipetalonema* sp. chez des puces récoltées sur les renards du sud-ouest de la France. Ann Parasit Hum Comp 49: 123-125.
52. Bain O, Ranque P (1974) Une nouvelle Filaire de Lézard *Befilaria africana* n. sp. ; appartenance de ce genre aux Oswaldofilariinae. Bull Mus natn Hist nat 3ème série 208: 159-167.
53. Bain O (1974) Description de nouvelles Filaires Oswaldifilariinae de Lézards sud-américains ; hypothèse sur l’évolution des Filaires de Reptiles. Bull Mus Natl Hist Nat 3 sér Zool 208 (138): 169-200.
54. Bain O 1974) Développement larvaire de Dipetalonema dessetae, Filaire de Rongeur entretenue au laboratoire. Ann Parasit Hum Comp 49: 457-466
55. Bain O, Durette-Desset MC, De Leon R (1974) Onchocercose au Guatemala; l’ingestion des microfilaires par Simulium ochraceum et leur passage dans l’hémocèle de ce vecteur. Ann Parasit Hum Comp 49: 467-487
56. Bussiéras J, Amégée E, Bain O (1974) Les onchocercoses des bovins togolais à *O. dukei* et *O. dermata*. Rev Elev Med Vét Pays Trop 27: 89-194.
57. Bain O, Schulz-Key H (1974) Les onchocerques du Cerf européen: redescription d’*O. flexuosa* (Wedl, 1856) et description d’*O. tubingensis* n. sp. et d’*O. tarsicola* n. sp. Tropenmed Parasit 25: 437-449.
58. Bain O, Schulz-Key H (1974) Une filaire intradermique chez le Cerf européen : *Cutifilaria wenki* n. gen. n. sp. (Onchocercinae). Tropenmed Parasit 25: 450-453.
59. Holler C, Bain O, Lapierre J (1974) Utilisation de *Dipetalonema dessetae* Bain, 1973, nouvelle Filaire de Rongeur (*Proechimys* sp.) comme antigène dans le diagnostic des filarioses humaines par la méthode d’immuno-fluorescence. CR Soc Biol 168: 449-452.
60. Prod’Hon J, Bain O (1974) *Madochotera landauae* n. sp, nouvelle Filaire de Grenouille malgache. Bull Mus Natl Hist Nat 3 sér Zool 232 (156): 813-817.
61. Bain O, Sulahian A (1975) Trois nouvelles Filaires du genre *Oswaldofilaria* chez des Lézards sud-américains; essai de classification des Oswaldofilariinae. Bull Mus Natl Hist Nat 3 sér Zool 232 (156): 827-841.
62. Bain O, Prod’Hon J (1974) Homogénéité des Filaires de Batraciens des genres *Waltonella*, *Ochoterenella* et *Madochotera*; création des Waltonellinae n. sub. fam. Ann Parasit Hum Comp 49: 721-739.
63. Bain O, Chabaud AG (1975) Développement chez des moustiques de trois Filaires de Lézards sud-américains du genre *Oswaldofilaria*. Ann Parasit Hum Comp 50: 209-221.
64. Bain O, Chaniotis BN (1975) *Befilaria puertoricensis* n. sp. nouvelle filaire Oswaldofilariinae d’Iguanidae aux Caraïbes (Puerto Rico). Bull Mus Natl Hist Nat 3 sér Zool 281 (191): 1-5.
65. Bain O, Chabaud AG (1975) Le mécanisme assurant la régulation de la traversée de la paroi stomacale du vecteur par les microfilaires (*Dipetalonema dessetae*- *Aedes aegypti*). CR Acad Sci sér D 281: 1199-1202.
66. Bain O (1975) Redescription de cinq espèces d’Onchocerques. Ann Parasit Hum Comp 50: 763-788.
67. Mougey Y, Bain O (1976) Passage des microfilaires dans l’hémocèle du vecteur; modèles stochastiques appropriés à diverses hypothèses sur les mécanismes de la limitation. Ann Parasit Hum Comp 51: 95-110.
68. Bain O (1976) Traversée de la paroi stomacale du vecteur par les microfilaires. Techniques utilisées, importance épidémiologique. WHO. FIL/75.138. Bull OMS 54: 397-401.
69. Anderson RC, Bain O (1976) Keys to genera of the order Spirurida. Part. 3. Diplotriaenoidea, Aproctoidea and Filarioidea. In: Anderson RC, Chabaud AG, Willmott S, editors. CIH Keys to the Nematode parasites of Vertebrates. N°3. pp 59-116.
70. Schulz-Key H, Bain O, Wenk P (1976) Untersuchungen über die Filarien der Cerviden in Sud-deutschland. 4. *Onchocerca garmsi* Bain und Schulz-Key, 1976, eine subkutane Filarie des Rothrirsches (*Cervus elaphus*). Tropenmed Parasit 27: 229-232.
71. Bain O, Philippon B, Séchan Y, Cassone J (1976) Corrélations entre le nombre de microfilaires ingérées et l’épaisseur de la membrane péritrophique du vecteur dans l’Onchocercose de savane africaine. CR Acad Sci sér D 283: 391-392.
72. Bain O, Bussiéras J, Amégée E (1976) Compléments à l’étude de deux onchocerques de Bovins africains. Ann Parasit Hum Comp 51: 461-471.
73. Bain O, Schulz-Key H (1976) Une quatrième espèce d’Onchocerque *O. garmsi* n. sp. chez le Cerf européen. Tropenmed Parasit 27: 474-478.
74. Bain O, Muller RL, Khamis Y, Guilhon J, Schilhorn Van Veen T (1976) *Onchocerca raillieti* n. sp. (Filarioidea) chez l’Ane domestique en Afrique. J Helminth 50: 287-293.
75. Chabaud AG, Bain O (1976) La lignée *Dipetalonema*. Nouvel essai de classification. Ann Parasit Hum Comp 51: 365-397.
76. Bain O, Ramachandran CP, Petter F, Mak JW (1977) Description d’*Onchocerca dewittei* n. sp. (Filarioidea) chez *Sus scrofa* en Malaisie. Ann Parasit Hum Comp 52: 471-479.
77. Petit G, Bain O, Spitalier-Kaveh H (1977) Facteurs favorables à la transmission de la filaire de laboratoire *Dipetalonema dessetae*. Ann Parasit Hum Comp 52, 585-586.
78. Bain O, Quentin JC (1977) Développement de *Dipetalonema (A.) weissi*, filaire de Macroscélide chez un ornithodore. Ann Parasit Hum Comp 52: 569-575.
79. Bain O, Chabaud AG (1977) Le mécanisme assurant la régulation de la traversée de la paroi stomacale du vecteur *Aedes aegypti* par les microfilaires de *Dipetalonema dessetae*. Ann Parasit Hum Comp 52: 84-86.
80. Bain O, Shoho C (1978) Sur deux Filaires d’Ongulés, en Malaisie. Ann Parasit Hum Comp 53: 93-100.
81. Bain O, Denké AM, Amégée Y, Chabaud AG (1977) Les Onchocerques des bovins au Togo. Les microfilaires et leurs distributions. Ann Univ Bénin Togo 3: 117-123.
82. Bain O (1978) Développement en Camargue de la Filaire du chien *Dirofilaria repens* Railliet et Henry, 1911, chez les *Aedes* halophiles. Bull Mus Natl Hist Nat 3 sér Zool. 510:19-27.
83. Bain O, Muller R (1978) Examen de quelques spécimens d’Onchocerques animales récoltées par le Dr. Le Roux en Afrique orientale. Ann Parasit Hum Comp 53: 311-313.
84. Bain O, Petit G (1978) Redescription du stade infestant d’*Onchocerca cervicalis* R. et H, 1910. Ann Parasit Hum Comp 53: 315-318.
85. Bain O, Chabaud AG, Landau I (1978) Trois nouvelles Onchocerques chez des Céphalophes du Gabon. Ann Parasit Hum Comp 53: 403-419.
86. Bain O, Petit G, Poulain B (1978) Validité de deux espèces *Onchocerca lienalis* et *O. gutturosa* chez les bovins. Ann Parasit Hum Comp 53: 421-430.
87. Bain O, Chabaud AG (1978) *Litomosa wite* Krepkogorskaya, 1933 (Nematoda): proposed correction to *Litomosa viteae*. Bull Zool nomencl 35: 51-54.
88. Hira HPR, Bain O, Petit G, Patel BG, Muller R (1978) Une Onchocerque des bovins en Afrique de l’Ouest, *O. dukei*, retrouvée en Zambie. Ann Parasit Hum Comp 53: 309-310.
89. Denké AM, Bain O (1978) Données sur le cycle d’*Onchocerca ochengi* chez *Simulium damnosum* s. l. au Togo. Ann Parasit Hum Comp 53: 757-760.
90. Bain O, Beveridge I (1979) Redescription d’*Onchocerca gibsoni* C. et J, 1910. Ann Parasit Hum Comp 54: 69-80.
91. Bain O, Beveridge I (1979) Redescription du mâle d’*Onchocerca lienalis* (Filarioidea, Nematoda). Ann Parasit Hum Comp 54: 117-118.
92. Tibayrenc M, Bain O, Ramachandran CP (1979) Deux nouvelles *Litomosa* (Filarioidea) de Chauve-souris. Bull Mus Natl Hist Nat 4 sér sect A 1: 183-189.
93. Bain O, Tibayrenc M, Mak JW (1979) Deux espèces de *Breinlia* (Filarioidea) chez un Ecureuil en Malaisie. Bull. Bull Mus Natl Hist Nat 4 sér sect A 1: 191-197.
94. Bain O, Kim DC, Petit G (1979) Diversité spécifique des Filaires du genre *Waltonella* coexistant chez *Bufo marinus*. Bull Mus Natl Hist Nat 4 sér sect A 1: 199-212.
95. Bain O, Rehbinder C, Petit G (1979) Présence d’*Onchocerca tarsicola* Bain et Schulz-Key 1974, chez le Renne. Ann Parasit Hum Comp 54: 263-265.
96. Bain O, Chabaud AG (1979) Sur les Muspiceidae (Nematoda Dorylaimina). Ann Parasit Hum Comp 54: 207-225.
97. Bain O (1979) Transmission de l’Onchocerque bovine, *Onchocerca gutturosa* par Culicoides. Ann Parasit Hum Comp 54: 483-488.
98. Doucet MM, Laumond C, Bain O (1979) *Empidomermis riouxi* n. sp. (Nematoda, Mermithidae) parasite d’*Aedes (Ochlerotatus) detritus* (Haliday, 1833). Ann Parasit Hum Comp 54: 341-351.
99. Moorhouse DE, Bain O, Wolf G (1979) *Josefilaria mackerrasae* gen. et sp. nov. (Nematoda : Filarioidea), parasite de la chauve-souris *Macroderma gigas* Dobson. Ann Parasit Hum Comp 54: 645-652.
100. Petit G, Bain O, Ratanaworabhan N, Yenbutra S, Chabaud AG (1980) Développement larvaire d’une filaire parasite d’un écureuil Petauristinae chez des *Aedes*. Ann Parasit Hum Comp 55: 147-152.
101. Bain O, Petit G, Berteaux S (1980) Description de deux nouvelles filaires du genre *Litomosoides* et de leurs stades infestants. Ann Parasit Hum Comp 55: 225-237.
102. Bain O, Chabaud AG, Wanantasamruad P, Nabhitabhata J (1980) *Onchocerca sweetae* chez le Buffle en Thaïlande. Ann Parasit Hum Comp 55: 253-259.
103. Chabaud AG, Seureau C, Beveridge I, Bain O, Durette-Desset MC (1980) Sur les Nématodes Echinonematinae. Ann Parasit Hum Comp 55: 427-443.
104. Bain O (1980) Deux filaires du genre *Eufilaria* chez le Merle : développement chez *Culicoides nubeculosus*. Ann Parasit Hum Comp 55: 583-590.
105. Baker MR, Bain O (1981) *Spinicauda voltaensis* n. sp. (Nematoda: Heterakoidea) from a toad of Upper Volta, Africa. System Parasitol 2: 139-144.
106. Chabaud AG, Bain O (1981) Description de *Spirobakerus weitzeli* n. g. n. sp. et remarques sur les Nématodes Spirocercidae. Ann Parasit Hum Comp 56: 73-80.
107. Bain O, Petit G, Kozek WJ, Chabaud AG (1981) Sur les filaires Splendidofilariinae du genre *Aproctella*. Ann Parasit Hum Comp 56: 95-105.
108. Bain O, Petit G, Ratanaworabhan N, Yenbutra S, Chabaud AG (1981) Une nouvelle filaire d’écureuil en Thaïlande, *Breinlia (B.) manningi* n. sp. et son développement chez *Aedes*. Ann Parasit Hum Comp 56: 193-201.
109. Chabaud AG, Bain O (1981) *Quentius kozeki* n. g, n. sp, Nématode rictulaire parasite d’un Marsupial américain. Ann Parasit Hum Comp 56: 173-178.
110. Bain O (1981) Les espèces du genre *Onchocerca* et principalement *O. volvulus*, envisagées du point de vue épidémiologique et phylogénique. Ann Soc Belge Méd Trop 61: 225-231.
111. Baker MR, Bain O (1981) *Falcaustra belemensis* n. sp. (Nematoda, Kathlaniinae) from the lizard *Neusticurus bicarinatus* L. (Teiidae) of Brazil. Bull Mus Natl Hist Nat 4 sér sect A 3: 117-122.
112. Denke AM, Bain O (1981) Deux nouvelles Onchocerques nodulaires chez des Bovidés sauvages en Haute-Volta. Ann Parasit Hum Comp 56: 339-347.
113. Bain O (1981) Filariids and their evolution. Parasitology, 82: 161-174.
114. Bain O, Nasher K (1981) Redescription de l’Onchocerque du dromadaire *O. fasciata* R. et H, 1910. Ann Parasit Hum Comp 56: 401-406.
115. Bain O (1981) Le genre *Onchocerca*: hypothèses sur son évolution et clé dichotomique des espèces. Ann Parasit Hum Comp 56: 503-526.
116. Bain O (1981) Redescription du stade infestant de la filaire *Parafilaria bovicola*: affinités du genre avec les *Thelazia*. Ann Parasit Hum Comp 56: 527-530.
117. Bain O (1982) Notes et discussions sur les oiseaux et leurs parasites. I. Spectres d’hôtes des Aprocta (Filaires ovipares). Mém Mus Natl Hist Nat sér A Zool 123: 270-271
118. Bain O, Mawson PM (1982) On some oviparous filarial Nematodes mainly from Australian birds. Rec S Aust Mus 18: 265-284.
119. Hugot JP, Bain O, Cassone J (1982) Insémination traumatique et tube de ponte chez l’Oxyure parasite du lapin domestique. CR Acad Sci sér III 294: 707-710.
120. Anderson RC, Bain O (1982) Keys to genera of the superfamilies Rhabditoidea, Dioctophymatoidea, Trichinelloidea and Muspiceoidea. In: Anderson RC, Chabaud AG, Willmott S, editors. CIH Keys to the nematode parasites of vertebrates, N°9. pp 1-26.
121. Beaucournu JC, Bain O (1982) *Ctenocephalides chabaudi* sp. n. (Siphonaptera, Pulicidae), puce nouvelle de la forêt primaire du Gabon. Ann Parasit Hum Comp 57: 165-168.
122. Bain O, Wertheim G (1981) Helminthes d’oiseaux et de mammifères d’Israël. IX. Compléments morphologiques sur quelques *Capillaria* (Nematoda Trichinelloidea). Bull Mus Natl Hist Nat 4 sér sect A 3: 1061-1075.
123. Bain O, Kouyate B, Baker M (1982) Nouvelles données sur les Oswaldofilariinae (Filarioidea, Nematoda). Bull Mus Natl Hist Nat 4 sér sect A 4: 61-70.
124. Richard-Lenoble D, Kombila M, Bain O (1982) Foyer de filariose humaine au Gabon à microfilaire dermique indifférenciable de *Microfilaria rodhaini*. Ann Parasit Hum Comp 57: 506.
125. Bain O, Vassiliades G, Delbove P (1982) Une nouvelle Onchocerque parasite de bovin domestique au Sénégal. Ann Parasit Hum Comp 57: 587-591.
126. Bain O, Baker M, Chabaud AG (1982) Nouvelles données sur la lignée *Dipetalonema* (Filarioidea, Nematoda). Ann Parasit Hum Comp 57: 593-620.
127. Bain O, Aeschlimann A, Chatelanat P (1982) Présence, chez des tiques de la région de Genève, de larves infestantes qui pourraient se rapporter à la filaire de chien *Dipetalonema grassii*. Ann Parasit Hum Comp 57: 643-646.
128. Bain O, Kouyate B, Cassone J (1983) *Edesonfilaria cynocephali* n. sp, filaire parasite de Dermoptère en Malaisie. Ann Parasit Hum Comp 58: 185-191.
129. Bain O, Nikander S (1983) Un nématode aphasmidien dans les capillaires de l’oreille du Renne *Lappnema auris* n. gen. n. sp. (Robertdollfusidae). Ann Parasit Hum Comp 58: 383-390.
130. Chabaud AG, Bain O, Hugot JP, Rausch RL, Rausch VL (1983) Organe de de Man et insémination traumatique. Rev Nématol 6: 127-131.
131. Hugot JP, Bain O, Cassone J (1983) Sur le genre *Passalurus* (Oxyuridae : Nematoda) parasite de Léporidés. System Parasitol 5: 305-316.
132. Bain O, Purnomo, Dedet JP (1983) Une nouvelle filaire *Chabfilaria jonathani* n. gen. n. sp, Onchocercidae parasite de Xénarthre. Ann Parasit Hum Comp 58: 583-591.
133. Petit G, Bain O, Gomes AF, Touratier L (1983) *Piratuboides huambensis* n. sp, filaire Oswaldofilariinae parasite de lézard en Afrique australe. Bull Mus Natl Hist Nat 4 sér sect A 5: 743-747.
134. Vassiliades G, Delbove P, Bain O (1983) Onchocercoses bovines au Sénégal. Note préliminaire. Rev Elev Med Vet Pays Trop 36: 351-353.
135. Chabaud AG, Navone GT, Bain O (1983) Description de *Mazzia bialata* n. sp, parasite de Dasypodidés. Attribution du genre aux Nématodes Spirocercidae. Bull Mus Natl Hist Nat 4 sér sect A 5:175-179.
136. Launay H, Deunff J, Bain O (1983) *Spilotylenchus arthurigen* n. sp. n. (Nematoda, Tylenchida: Allantonematidae), parasite de *Spilopsyllus cuniculi* (Dale, 1878) (Siphonaptera: Pulicidae). Ann Parasit Hum Comp 58: 141-150.
137. Bain O, Purnomo (1984) Description d’*Icosiella laurenti* n. sp, filaire de Ranidae en Malaisie et hypothèse sur l’évolution des Icosiellinae. Bull Mus Natl Hist Nat 4 sér sect A 6: 31-36.
138. Millet P, Bain O (1984) Une nouvelle filaire de la pie, *Eufilaria kalifai* n. sp. (Lemdaninae) et son développement chez *Culicoides nubeculosus*. Ann Parasit Hum Comp 59: 177-188.
139. Petit G, Bain O, Roussilhon C (1985) Deux nouvelles filaires chez un Singe, *Saimiri sciureus*, en Guyane. Ann Parasit Hum Comp 60: 65-81.
140. Bain O, Petit G, Gueye A (1985) Transmission expérimentale de *Monanema nilotica* El Bihari et coll, 1977, filaire à microfilaires dermiques parasite de muridés africains. Ann Parasit Hum Comp 60: 83-89.
141. Vuong Ngoc P, Bain O, Petit G, Chabaud AG (1985) Etude comparative des lésions cutanées et oculaires du muridé *Lemniscomys striatus* parasité par *Monanema* spp. et d’*Atherurus africanus* parasité par *Cercopithifilaria* sp. avec celles de l’onchocercose humaine. CR Acad Sci sér. III 301: 433-435.
142. Bain O, Petit G, Vuong NP, Chabaud AG (1985) Filaires de rongeurs favorables à l’étude expérimentale de l’onchocercose humaine. CR Acad Sci sér. III 301: 513-515.
143. Bain O, Dissanaike AS, Cross JH, Harinasuta C, Sucharit S (1985) Morphologie de *Wuchereria bancrofti* adulte et sub-adulte. Recherche de caractères différentiels entre les souches. Ann Parasit Hum Comp 60: 613-630.
144. Bain O, Petit G, Jacquet-Viallet P, Houin R (1985) *Cherylia guyanensis* n. gen. n. sp, filaire d’un marsupial sud-américain, transmise par tique. Ann Parasit Hum Comp 60: 727-737.
145. Bain O, Vuong NP, Petit G, Prod’Hon J, Ranque P, et al. (1986) Différences dans la localisation des microfilaires d’*O. volvulus* en savane et en forêt: implications cliniques éventuelles. Ann Parasit Hum Comp 61: 125-126.
146. Bain O, Petit G, Chabaud AG (1986) Une nouvelle filaire, *Cercopithifilaria roussilhoni* n. sp, parasite de l’Athérure au Gabon, transmise par tiques: hypothèse sur l’évolution du genre. Ann Parasit Hum Comp 61: 81-93.
147. Bain O, Denke AM (1986) Larves infestantes d’une filaire: (?) *Cercopithifilaria* sp. chez des tiques de bovins au Togo. Ann Parasit Hum Comp 61: 131-135.
148. Bain O, Petit G, Chabaud AG (1985) Nouvelles données sur la transmission des filaires. Bull Soc Path Exot 78: 756-762.
149. Vuong NP, Bain O, Petit G, Chabaud AG (1986) Etude anatomo-pathologique des lésions cutanées et oculaires de rongeurs infestés par *Monanema* spp. Intérêt pour l’étude de l’onchocercose humaine. Ann Parasit Hum Comp 61: 311-320.
150. Bain O, Bartlett C, Petit G (1986) Une filaire de muridés africains dans la paroi du côlon, *Monanema martini* n. sp. Ann Parasit Hum Comp 61: 465-472.
151. Bain O, Rehbinder C (1986) Nouvelle onchocerque, *Onchocerca alcis* n. sp, parasite de l’élan *Alces alces* en Suède. Ann Parasit Hum Comp 61: 447-455.
152. Bain O, Chabaud AG (1986) Atlas des larves infestantes de Filaires. Trop Med Parasit 37: 301-340.
153. Chabaud AG, Bain O, Landau I, Petit G (1986) La transmission des parasites par vecteurs hématophages: richesse des phénomènes adaptatifs. La Vie des Sciences, 3: 469-484.
154. Bain O, Petit G, Rosales-Loesener L (1986) Filaires de singes sud-américains. Bull Mus Natl Hist Nat 4 sér sect A 8:513-542.
155. Bain O, Vuong NP, Petit G, Chabaud AG, Capron A, et al. (1987) Modifications cutanées provoquées par une dose de DEC chez des rongeurs à microfilaires dermiques; intérêt de ces phénomènes pour comprendre la réaction de Mazzotti et la pathogénie de l’onchocercose humaine. CR Acad Sci sér III 304: 133-138.
156. Bartlett CM, Bain O (1987) New avian Filarioids (Nematoda: Splendidofilariinae): *Dessetfilaria guianensis* gen. n, sp. n, *Andersonfilaria africanus* gen. n, sp. n, and *Splendidofilaria chandenieri* sp. n. Proc Helminth Soc Wash 54: 1-14.
157. Bain O, Diagne M, Muller R (1987) Une cinquième filaire du genre *Dipetalonema*, parasite de singes sud-américains. Ann Parasit Hum Comp 62: 262-270.
158. Chabaud AG, Bain O, Landau I, Petit G (1987) Recent data on the transmission mechanisms of Filarioids and Plasmodia, controling the host-parasite equilibrium. Parasite Host Environment, 1: 174-184.
159. Prod’Hon J, Lardeux F, Bain O, Hebrard G, Prud’Hom JM (1987) Ivermectine et modalités de la réduction de l’infection des Simulies dans un foyer forestier de l’onchocercose humaine. Ann Parasit Hum Comp 62: 590-598.
160. Justine JL, Ferté H, Bain O (1987) Trois *Capillaria* (Nematoda) de l’intestin du lérot en France. Rapports avec un *Capillaria* de l’estomac du sanglier. Bull Mus Natl Hist Nat 4 sér sect A 9: 579-604.
161. Petit G, Bain O, Carrat C, de Marval F (1988) Développement de la filaire *Monanema martini* dans l’épiderme des tiques Ixodidae. Ann Parasit Hum Comp 63: 54-63.
162. Justine JL, Bain O (1987) *Capillaria petiti* n. sp. (Nematoda, Capillariinae), parasite du crapaud *Bufo marinus* (Amphibia) au Brésil. Bull Mus Natl Hist Nat 4 sér sect A 9: 815-828.
163. Bain O, Chandrasekharan SA, Partono F, Mak JW, Zheng HJ, et al. (1988) Discrimination de souches géographiques de *Brugia malayi* périodique par l’ornementation cuticulaire des mâles. Ann Parasit Hum Comp 63: 209-223.
164. Bain O, Wamae CN, Reid GDF (1988) Diversité des filaires du genre *Cercopithifilaria* chez les babouins au Kenya. Ann Parasit Hum Comp 63: 224-239.
165. Vuong PN, Bain O, Cabaret J, Petit G, Prod’Hon J, et al. (1988) Forest and savanna onchocerciasis: comparative morphometric histopathology of skin lesions. Tropen Med Parasit 39: 105-110.
166. Petit G, Bain O, Cassone J, Seureau C (1988) La filaire *Cercopithifilaria roussilhoni* chez la tique vectrice. Ann Parasit Hum Comp 63: 296-302.
167. Chabaud AG, Bain O, Poinar GO (1988) *Skrjabinelazia galliardi* (Nematoda, Seuratoidea): compléments morphologiques et cycle biologique. Ann Parasit Hum Comp 63: 278-284.
168. Bain O, Chabaud AG (1988) Un appareil favorisant l’accouplement des filaires: les renflements de la région antérieure du corps. Ann Parasit Hum Comp 63: 376-379.
169. Bain O, Chabaud AG, Georges AJ (1987) Nouvelle filaire du genre *Cercopithifilaria*, parasite d’un carnivore africain. Parassitologia 29: 63-69.
170. Laukamm-Josten U, Bain O (1988) The skin-dwelling microfilariae of *Monanema martini* in *Lemniscomys striatus* as potential drug screening model for onchocerciasis: midazolam effect in vitro. Acta Tropica 45: 373-377.
171. Van Waerebeke D, Chabaud AG, Bain O, Georges AJ (1988) Deux nouveaux Nématodes parasites de poissons de l’Oubangui. Bull Mus Natl Hist Nat 4 sér sect A 10: 519-527.
172. Bain O, Wamae CN, Reid GDF (1988) Description de *Cercopithifilaria verveti* n. sp, filaire sous-cutanée d’un cercopithèque au Kenya. Ann Parasit Hum Comp 64: 42-45.
173. Chartier C, Bain O, Nzymana S (1988) Note sur l’existence d’*Elaeophora poeli* (Vyburg, 1879) Railliet & Henry, 1912, chez le Zébu (*Bos indicus*) en Ituri (Haut-Zaïre). Ann Soc Belge Méd Trop 68: 353-356.
174. Diagne M, Petit G, Bain O (1989) Maintien d’une filaire chez la souris. CR Acad Sci sér III 309: 25-28.
175. Bain O, Petit G, Diagne M (1989) Etude de quelques *Litomosoides* parasites de rongeurs; conséquences taxonomiques. Ann Parasit Hum Comp 64: 251-256.
176. Poinar GO, Chabaud AG, Bain O (1989) *Rabbium paradoxus* sp. n. (Seuratidae: Skrjabinelaziinae) maturing in *Camponotus castaneus* (Hymenoptera: Formicidae). Proc Helm Soc Wash 56: 120-124.
177. Richard-Lenoble D, Kombila M, Bain O, Chandenier J, Mariotte O (1988) Filariasis in Gabon: human infections with *Microfilaria rodhaini*. Am J Trop Med Hyg 39: 91-92.
178. Diagne M, Petit G, Seureau C, Bain O (1989) Développement de la filaire *Litomosoides galizai* chez l’acarien vecteur. Ann Parasit Hum Comp 64: 478-488.
179. Takaoka H, Baba M, Bain O (1989) Natural infections of *Simulium bidentatum* (Diptera: Simuliidae) with larvae of *Onchocerca* spp, in relation to a human zoonotic onchocerciasis in Oita, Japan. Japan J Trop Med Hyg,17: 279-284.
180. Bain O, Petit G (1989) Ecologie de la transmission des filaires. Analyse expérimentale des rapports parasites-vecteurs. Bull Ecol 20: 281-288.
181. Takaoka H, Bain O (1990) Infections of blackflies (Diptera: Simuliidae) with three types of zoonotic *Onchocerca* larvae in Oita, Japan. Japan J Trop Med Hyg 18: 1-10.
182. Bain O, Camus D, Prod’Hon J (1990) Parasitology in France: Some aspects of the Present. Parasitol Today 6: 209-217.
183. Chabaud AG, Bain O (1990) Trois nouvelles filaires parasites d’oiseaux de forêts équatoriales humides africaines. Bull Mus Natl Hist Nat 4 sér sect A 12: 9-18.
184. Wanji S, Cabaret J, Gantier JC, Bonnand N, Bain O (1990) The fate of the filaria *Monanema martini* in two rodent hosts: recovery rate, migration and localization. Ann Parasit Hum Comp 65: 80-88.
185. Laukamm-Josten U, Bain O, Kremsner P (1989) The effect of the benzodiazepine antagonist flumazenil on microfilariae *Monanema martini* *in vitro*. Acta Tropica 47: 125-127.
186. Diagne M, Petit G, Liot P, Cabaret J, Bain O (1990) The filaria *Litomosoides galizai* in mites; microfilarial distribution in the host and regulation of the transmission. Ann Parasit Hum Comp 65: 193-199.
187. Bain O, Philipp M (1991) Animal models in the study of the phenomenon of parasitism: filariae and other parasites. Ann Parasit Hum Comp 66: 64-68.
188. Vuong PN, Wanji S, Sakka L, Klager S, Bain O (1991) The murid filaria *Monanema martini*: a model for onchocerciasis. Part I: Description of lesions. Ann Parasit Hum Comp 66: 109-120.
189. Post R, Bain O, Klager S (1991) Chromosome numbers in *Onchocerca dukei* and *O. tarsicola*. J Helminth 65: 208-210.
190. Petit G, Diagne M, Maréchal P, Owen D, Taylor D, et al. (1992) Maturation of the filaria *Litomosoides sigmodontis* in BALB/c mice; comparative susceptibility of nine other inbred strains. Ann Parasit Hum Comp 67, 144-150.
191. Bain O, Petit G, Paperna I, Finkelman S, Killick-Kendrick M (1992) A new filaria of a lizard transmitted by sandflies. Mem Inst Oswaldo Cruz Suppl 1 87: 21-29.
192. Bain O, Chabaud AG, Burger WP (1992) *Versternema struthionis* n. gen. n. sp, filaire archaïque à morphologie peu spécialisée. Ann Parasit Hum Comp 67: 141-143.
193. Vuong PN, Traore S, Wanji S, Diarabassouba S, Balaton A, et al. (1992) Ivermectin in human onchocerciasis: a clinical-pathological study of skin lesions before and three days after treatment. Ann Parasit Hum Comp 67: 194-196.
194. Maréchal P, Cabaret J, Petit G, Diagne M, Gasnier N, et al. (1993) Isoenzymatic diagnosis of *Litomosoides galizai* and *Litomosoides sigmodontis*. Ann Parasit Hum Comp 68: 61-62.
195. Bain O, Wahl G, Renz A (1993) *Onchocerca ramachandrini* n. sp. from the warthog in Cameroon. Ann Parasit Hum Comp 68: 139-143.
196. Chandre F, Petit G, Diagne M, Maréchal P, Bain O (1993) Effect of ivermectin on two filaria vector pairs: *Brugia malayi*-*Aedes aegypti*, *Litomosoides sigmodontis*-*Bdellonyssus bacoti*. Ann Parasit Hum Comp 68: 144-149.
197. Bain O, Renz A (1993) Infective larvae of a new species of Robertdollfusidae (Adenophorea, Nematoda) in the gut of *Simulium damnosum* in Cameroon. Ann Parasit Hum Comp 68: 182-184.
198. Vuong PN, Spratt D, Wanji S, Aimard L, Bain O (1993) Onchocerca-like lesions induced by the filarioid nematode *Cercopithifilaria johnstoni*, in its natural hosts and in the laboratory rat. Ann Parasit Hum Comp 68: 176-181.
199. Bain O, Petit G, Paperna I, Finkelman S, Killick-Kendrick M (1993) Filaires Splendidofilariinae de lézards: nouvelles espèces, redescription, cycle chez phlébotome. Syst Parasitol 26: 97-115.
200. Aimard L, Wanji S, Vuong PN, Petit G, Bain O (1993) Ophthalmological study of the lesions induced by the filarial worm with dermal microfilariae, *Monanema martini*, in its murid hosts. Curr Eye Res 12: 885-891.
201. Xie H, Bain O, Williams SA (1994) Molecular phylogenetic studies on filarial parasites based on 5S ribosomal spacer sequences. Parasite 1: 141-151.
202. Maréchal P, Petit G, Diagne M, Taylor DW, Bain O (1994) Use of the *Litomosoides sigmodontis* – mouse model in development of an Onchocerca vaccine. II – L. sigmodontis in the BALB/c mouse: vaccination experiments; preliminary immunological studies. Parasite S1 1: 31-32.
203. Aimard L, Wanji S, Vuong PN, Petit G, Bain O (1994) Clinical study of the ocular lesions induced by *Monanema martini* in its murid hosts. Parasite S1 1: 33.
204. Wanji S, Vuong PN, Gantier JC, Bougnoux ME, Breton B, et al. (1994) Drug trials with *Monanema martini*: effect on the adult worms, the dermal microfilariae and the natural murid host. Parasite S1 1: 33-34.
205. Wanji S, Gantier JC, Petit G, Rapp J, Bain O (1994) *Monanema martini* in its murid hosts: microfiladermia related to infective larvae and adult filariae. J Trop Parasit 45: 107-111.
206. Xie H, Bain O, Williams SA (1994) Molecular phylogenetic studies on *Brugia filariae* using HHA 1 repeat sequence. Parasite 1: 255-260.
207. Bain O, Wanji S, Vuong PN, Maréchal P, Le Goff L, et al. (1994) Larval biology of six filariae of the subfamily Onchocercinae in the vertabrate host. Parasite 1: 241-254.
208. Yagi K, Bain O, Shoho C (1994) Onchocerca suzukii n. sp. and *O. skrjabini* (=*O. tarsicola*) from a relict bovid, *Capricornis crispus*, in Japan. Parasite 1: 349-356.
209. Vuong PN, Wanji S, Prod’Hon J, Bain O (1994) Nodules sous-cutanés et lésions cutanées engendrées par diverses onchocerques chez des bovins africains. Revue Elev Méd Vét Pays Trop 47: 47-51.
210. Nozais JP, Bain O, Gentilini M (1995) Un cas de dirofilariose sous-cutanée à *Dirofilaria (Nochtiella) repens* avec microfilarémie en provenance de Corse. Bull Soc Path Exot 87: 183-185.
211. Chabaud A.G, Bain O (1994) The evolutionary expansion of the Spirurida. Intern. J Parasitol 24: 1179- 1201.
212. Bain O, Wanji S, Vuong PN, Petit G, Breton B, et al. (1994) Cardio-pulmonary location of lymphatic filariae. Lymphology 27: 385-388.
213. Wahl G, Bain O (1995) Development by injection in *Simulium damnosum* s. l. of two *Onchocerca* species from the warthog to infective larvae resembling type D larvae (Duke, 1967). Parasite 2: 55-62.
214. Basanez MG, Remme JHF, Alley ES, Bain O, Shelley AJ, et al. (1995) Density-dependent processes in the transmission of human onchocerciasis: relationship between the numbers of microfilariae ingested and successful larval development in the simuliid vector. Parasitology 110: 409-427.
215. Boussinesq M, Bain O, Chabaud AG, Gardon-Wendal N, Kamgno J, et al. (1995) Meningonemosis, a new filariid zoonosis of the cerebrospinal fluid of a man probably caused by *Meningonema peruzzi*, a parasite of the central nervous system of Cercopithecidae. Parasite 2: 173-176.
216. Boomker J, Bain O, Chabaud A, Kriek NPJ (1995) *Stephanofilaria thelazioides* n. sp. (Nematoda: Filariidae) from a hippopotamus and its affinities with the species parasite of the African black rhinoceros. Syst Parasitol 32: 205-210.
217. Bain O, Moisson P, Huerre M, Landsoud-Soukate J, Tutin C (1995) Filariae from a wild gorilla in Gabon with description of a new species of *Mansonella*. Parasite 2: 315-322.
218. Takaoka H, Aoki C, Bain O, Ogata K, Baba M (1995) Investigation of *Culicoides* (Diptera: Ceratopogonidae) in relation to the transmission of bovine *Onchocerca* and other filariae in central Kyushu, Japan. Parasite 2: 367-372.
219. Maréchal P, Le Goff L, Petit G, Diagne M, Taylor DW, et al. (1996) The fate of the filaria *Litomosoides sigmodontis* in susceptible and naturally resistant mice. Parasite 3: 25-31.
220. Takaoka H, Bain O, Tajimi S, Kashima K, Nakayama I, et al. (1996) Second case of zoonotic *Onchocerca* infection in a resident of Oita in Japan. Parasite 3: 179-182.
221. Bain O, Van Der Lugt L, Kazadi IM (1996) *Stephanofilaria boomkeri* n. sp, as a cause of severe skin disease in pigs in Zaire. Parasite 4: 377-381.
222. Gasser R, Le Goff L, Petit G, Bain O (1997) Rapid delineation of closely-related filarial parasites using genetic markers in spacer rDNA. Acta Tropica 62: 143-150.
223. Maréchal P, Le Goff L, Hoffmann W, Rapp J, Oswald I, et al. (1997) Immune response to the filaria *Litomosoides sigmodontis* in susceptible and naturally resistant mice. Parasite Immunol 19: 273-279.
224. Le Goff L, Maréchal P, Petit G, Taylor DW, Hoffmann W, et al. (1997) Early reduction of the recovery rate following immunization with irradiated infective larvae in a filaria mouse system. TropMed Int Health 2: 1170-1174.
225. Breton B, Diagne M, Wanji S, Bougnoux ME, Chandre F, et al. (1997) Ivermectin and moxidectin: resistance of the filaria *Monanema martini*; inhibition of the insemination of *Litomosoides sigmodontis*. Parassitologia 39: 19-28.
226. Touré F, Kassambara L, Williams S, Millet P, Bain O, et al. (1997) Human occult loiasis improvement in diagnostic sensitivity by the use of nested polymerase chain reaction. Am J Trop Med Hyg 59: 144-149.
227. Touré F, Egwang P, Whal G, Millet P, Bain O, et al. (1997). Species-specific sequence in the repeat 3 region of gene encoding a putative Loa loa allergen: a diagnostic tool for occult loiasis. Am J Trop Med Hyg 56: 57-60.
228. Bain O, Wanji S, Enyong P, Petit G, Noireau F, et al. (1998). New features on the moults and morphogenesis of the human filaria *Loa loa* using rodent hosts. Consequences. Parasite 5: 37-46.
229. Uni S, Suzuki Y, Chiba H, Katsumi A, Takaoka H, et al. (1998) An *Onchocerca* species from cattle on Kyushu Island is *O. suzukii*, a transfuge parasite from the Japanese endemic bovid, *Capricornis crispus*. Japan J Trop Med Hyg 26: 157-160.
230. Pisanu, Bain O (1999) *Aonchotheca musimon* n. sp. (Nematoda: Capillariinae) from the mouflon *Ovis musimon* in the sub-antarctic Kerguelen archipelago, with comments on the relationships with *A. bilobata* (Bhalerao, 1933) Moravec, 1982 and other species of the genus. Syst Parasitol 43: 17-24.
231. Gautret P, Bain O, Gicquel JJ, Hue B, Kauffmann-Lacroix C, et al. (1999) Localisation sous-conjonctivale d’une femelle adulte de *Wuchereria banacrofti*. Bull Soc Path Exot 92: 104-106.
232. Rouhette H, Marty P, Zur C, Bain O, Fenollar S, et al. (1999) Ocular filariasis: not strictly tropical. Ophtalmologica 213: 206-208.
233. Panaitescu D, Preda A, Bain O, Vasile-Bugarin AC (1999) Four cases of human filariosis due to *Setaria labiatopapillosa* found in Bucharest, Romania. Roum Arch Microbiol Immunol 58: 203-207
234. Le Goff L, Martin C, Oswald IP, Vuong PN, Petit G, et al. (2000) Vaccination with irradiated larvae in the filarial model *Litomosoides sigmodontis*-BALB/c mice. Parasite biology and host immune response. Parasitology 120: 271-280.
235. Ungeheuer MN, Morelli A, Elissa N, Georges AJ, Debré P, et al. (2000) Cellular responses to *Loa loa* experimental infection in mandrills (*Mandrillus sphinx*) vaccinated with irradiated infective larvae. Parasite Immunol 22: 173-183.
236. Martin C, Le Goff L, Ungeheuer MN, Vuong PN, Bain O (2000) Drastic reduction of a filarial infection in eosinophilic IL-5 transgenic mice. Infect Immunity 68: 3651-3656.
237. Hoffmann W, Petit G, Schulz-Key H, Taylor DW, Bain O, et al. (2000) *Litomosoides sigmodontis* in mice: reappraisal of an old model for filarial research. Parasitol Today 16: 387-388.
238. Diagne M, Diouf M, Lochouarn L, Bain O (2000) *Trichosomoides nasalis* Biocca et Aurizi, 1961 et *T. spratti* n. sp. (Nematoda: Trichinelloidea), parasites des fosses nasales de muridés. Parasite 7: 215-220.
239. Hering-Hagenbeck S, Boomker J, Petit G, Killick-Kendrick M, Bain O (2000) Description and life cycle of *Madathamugadia hiepei* n. sp. (Splendidofilariinae: Nematoda), a parasite of a South African gecko. Syst Parasitol 47: 207-213.
240. Hering-Hagenbeck S, Boomker J, Bain O (2001) *Paraspirura bettinae* n. sp.parasitic in reptiles; relationships with *Protospirura* and *Spirura*, parasitic in mammals from a South African skink with comments on spirurid nematodes from saurians and mammals. J Parasitol 87: 838-844.
241. Martin C, Al-Qaoud K, Ungeheuer MN, Paehle K, Vuong PN, et al. (2001) IL-5 is essential for vaccine induced protection and for resolution of primary infection in murine filariasis. Med Microbiol Immunol 189: 67-74.
242. Guerrero R, Bain O (2001) The New World filarial genus *Molinema* Freitas & Lent, 1939 (Nematoda: Onchocercidae), with a descrition of four new species parasitic in the Echimyidae (Rodentia). Syst Parasitol 48: 203-221.
243. Notarnicola J, Bain O, Navone G (2001) Two new species of *Litomosoides* (Nematoda: Filarioidea) in sigmodontines (Rodentia: Muridae) from Rio de la Plata marshland, Argentina. J Parasitol 86: 1318-1325.
244. Volkmann L, Saeftel M, Bain O, Fischer K, Fleischer B, et al. (2001) Interleukin-4 is essential for the control of microfilariae in murine infection with the filaria *Litomosoides sigmodontis*. Infect Immunity 69: 2950-2956.
245. Uni S, Bain O, Takaoka H, Miyashita M, Suzuki Y (2001) *Onchocerca dewittei japonica* n. subsp, a common parasite from wild boar in Kyushu Island, Japan. Parasite 8: 215-222.
246. Takaoka H, Bain O, Uni S, Korenaga M, Tada K,et al. (2001) Human infection with *Onchocerca dewittei japonica*, a parasite from wild boar in Oita, Japan. Parasite 8: 261-263.
247. Uni S, Suzuki Y, Baba M, Mitani N, Takaoka H, et al. (2001) Coexistence of five *Cercopithifilaria* species in the Japanese rupricaprine bovid, *Capricornis crispus*. Parasite 8: 197-213.
248. Martin C, Saeftel M, Vuong PN, Babayan S, Fischer K,et al. (2001) B-cell deficiency suppresses vaccine-induced protection against murine filariasis but does not increase the recovery rate for primary infection. Infect Immunity 69: 7067-7073.
249. Notarnicola J, Bain O, Navone G (2002) *Litomosoides anguyai* n. sp. (Nematoda: Onchocercidae) from *Oxymycterus misionalis* (Rodentia: Muridae) in the rain forest of Misiones, Argentina. Syst Parasitol 52: 129-135.
250. Bain O, Babayan S, Gomes J, Guerrero R (2002) First account on the larval biology of a *Litomosoides filaria*, from a bat. Parassitologia 44: 89-92.
251. Guerrero R, Martin C, Gardner SL, Bain O (2002) New and known species of *Litomosoides* (Nematoda: Filarioidea): important adult and larval characters and taxonomic changes. Comp Parasitol 69: 177-195.
252. Baneth G, Volansky Z, Anug Y, Favia G, Bain O, et al. (2002) *Dirofilaria repens* infection in a dog: diagnosis and treatment with melarsomine and doramectin. Vet Parasitol 105: 173-178.
253. Bain O, Uni S, Takaoka H (2002) A synthetic look at a twenty years old taxon, *Cercopithifilaria* ; its probable evolution. Proceedings of the 10th International 5, 194, Congress of Parasitology-ICOPA X: Symposia, Workshops and contributor papers, Vancouver (Canada) August 4-9, Monduzzi Editore, 365-368.
254. Bain O, Kusaladharma PIT, Weerasooriya MV, Ihalamulla R, Dissanaike AS (2002) An immature filarial worm, probably *Wuchereria bancrofti*, from the anterior chamber of the eye in a patient from Sri Lanka. Parasite 9: 284-282.
255. Uni S, Bain O, Takaoka H, Fujita H, Suzuki Y (2002) Diversification of *Cercopithifilaria* species in Japanese wild ruminants with description of two new species. Parasite 9: 293-304.
256. Wanji S, Tendongfor N, Vuong PN, Enyong P, Bain O (2002) The migration and localisation of *Loa loa* infective and fourth stage larvae in normal and immunosuppressed rodents. Ann Trop Med Parasitol 96: 823-830.
257. Bain O (2002) Evolutionary relationships among filarial nematodes. In: Klei TR, Rajan TV, editors. World Class Parasites: Volume 5, The Filaria. pp21-29.
258. Takaoka H, Choochote W, Aoki C, Fukuda M, Bain O (2003) Black flies (Diptera : Simuliidae) attracted to humans and water buffalos and natural infections with filaria larvae, probably *Onchocerca* sp, in northern Thailand. Parasite 10: 3-8.
259. Bain O, Guerrero R (2003) *Bisbalia vossi* n. g, n. sp. (Nematoda: Onchocercidae), a filarial worm from a geomyoid rodent, *Heteromys anomalus,* in Venezuela. Syst Parasitol 54: 145-156.
260. Volkmann L, Bain O, Saeftel M, Specht S, Fischer K, et al. (2003) Murine filariasis: interleukin-4 and interleukin-5 lead to containment of different worm developmental stages. Med Microbiol Immunol 192: 23-31.
261. Buttner DW, Wanji S, Bazzochi C, Bain O, Fischer P (2003) Obligatory symbiotic *Wolbachia* endobacteria are absent from *Loa loa*. Filaria J 2(1):10.
262. Bain O, Guerrero R, Rodriguez B, Babayan S, Jouvenet N (2003) Examination of type material of *Litomosoides* spp. (Filarioidea: Onchocercidae), parasites from bats; taxonomic consequences. Parasite 10: 211-218.
263. Guerrrero R, Martin C, Bain O (2003) *Litomosoides yutajensis* n. sp, first record of this filarial genus in a mormoopid bat. Parasite 10: 219-225.
264. Fukuda M, Choochote W, Bain O, Aoki C, Takaoka H (2003) Natural infections with filarial larvae in two species of black flies (Diptera: Simuliidae) in Northern Thailand. Jap J Trop Med Hyg 31: 99-102.
265. Bain O, Babayan S (2003) The behaviour of filariae: morphological and anatomical signatures of their life style within the arthropod and vertebrate hosts. Filaria J 2(1):16.
266. Babayan S, Ungeheuer MN, Martin C, Attout T, Belnoue E, et al. (2003) Resistance and susceptibility to filarial infection with *Litomosoides sigmodontis* are associated with early differences in parasite development and in localized immune reactions. Infect Immunity 71: 6820-6829.
267. Casiraghi M, Bain O, Guerrero R, Martin C, Pocacqua V, et al. (2004) Mapping the presence of *Wolbachia pipientis* on the phylogeny of filarial nematodes: evidence for symbiont loss during evolution. Intern J Parasitol 34: 191-203.
268. Lhermitte-Vallarino N, Bain O (2004) Morphological and biological study of *Rhabdias* spp. (Nematoda) from African chameleons with description of a new species. Parasite 11: 15-31.
269. Uni S, Bain O, Takaoka H (2004) Affinities between *Cutifilaria* (Nematoda: Filarioidea), parasites of deer, and *Mansonella* as seen in a new onchocercid, *M. (C.) perforata* n. sp, from Japan. Parasite 11: 131-140.
270. Takaoka H, Bain O, Uni S, Korenaga M, Kozek WJ, et al. (2004) Zoonotic onchocerciasis caused by a parasite from wild boar in Oita, Japan: a comprehensive analysis of morphological characteristics of the worms for its diagnosis. Parasite 11: 285-292.
271. Diagne M, Vuong PN, Duplantier JM, Ba K, Thirion-Lochouarn L, et al. (2004) Histological study of *Trichomosoides nasalis* (Nematoda: Trichinelloidea) in the nasal cavities of the murid *Arvicanthis niloticus*, with associated pathology. Parasite 11: 351-358.
272. Attout T, Babayan S, Hoerauf A, Taylor DW, Kozek WJ, et al. (2004) Blood-feeding in the young adult filarial worms *Litomosoides sigmodontis*. Parasitology 130: 421-428.
273. Babayan S, Attout T, Specht S, Hoerauf A, Snounou G, et al. (2005) Increased early local immune responses and altered worm development in high dose infections of mice susceptible to the filaria *Litomosoides sigmodontis*. Med Microbiol Immunol 194: 151-162.
274. Takaoka H, Yanag T, Daa T, Anzai S, Aoki C, et al. (2005) An *Onchocerca* species of wild boar found in the subcutaneous nodule of a resident of Oita, Japan. Parasitol Int 54: 91-93.
275. Bain O, Attout T, Babayan S, Kozek W, Martin C (2005) Biology of filariae in the vertebrate host: *Litomosoides sigmodontis* casts light on several 'grey areas'. Editorial Medimond-International Proceedings, Monduzzi Editore.
276. Lhermitte-Vallarino N, Bain O, Deharo E, Bertani S, Voza T, et al. (2005) A new Rhabdiasid nematode, *Chabirenia cayennensis* n. g, n. sp, parasitic in the glands of the buccal mucosa of a South American saurian. Syst Parasitol 62:151-160.
277. Babayan S, Attout T, Vuong PN, Le Goff L, Gantier JC, et al. (2005) The subcutaneous movements of filarial infective larvae are impaired in vaccinated hosts in comparison to primary infected hosts. Filaria J 4:3.
278. Beytut E, Akca A, Bain O (2005) Teat onchocercosis in cows with reference to prevalence, species involved and pathology. Res Vet Sci 78: 45-51.
279. Fukuda M, Bain O, Aoki C, Otsuka Y, Takaoka H (2005) Natural infections of *Simulium (Nevermannia) uchidai* (Diptera: Simuliidae) with infective filarial larvae, probably from a bird, in Oita, Japan. Med Entomol Zool 56: 93-98.
280. Agatsuma T, Iwagami M, Uni S, Takaoka H, Katsumi A, et al. (2005) Molecular phylogenetic relationships among seven Japanese species of *Cercopithifilaria*. Parasitol Int 54: 195-199.
281. Ruiz de Ybanez MR, Mertinez-Carrasco C, Martinz JJ, Ortiz JM, Attout T, et al. (2006) *Dirofilaria immitis* in an African lion (*Panthera leo*). Vet Rec 158: 240-242.
282. Martin C, Bain O, Jouvenet N, Raharimanga V, Robert V, et al. (2006) First report of *Litomosa* spp. (Nematoda: Filarioidea) from Malagasy bats; review of the genus and relationships between species. Parasite 13: 3-10.
283. Guerrero R, Bain O, Attout T, Martin C (2006) The infective larva of *Litomosoides yutajensis* Guerero et al, 2003 (Nematoda: Onochcercidae), a Wolbachia-free filaria from bat. Parasite 13: 127-130.
284. Babayan S, Attout T, Harris A, Taylor M, Le Goff L, et al. (2006) Vaccination against filarial nematodes with irradiated larvae provides long-term protection against the third larval stage but not against subsequent life cycle stages. Int J Parasitol 36: 903-914.
285. Uni S, Bain O, Agatsuma T, Katsumi A, Bba M, et al. (2006) New filaria nematode from Japanese serows (*Naemorhedus crispus*: Bovidae) close to parasites from elephants. Parasite 13: 193-200.
286. Nfon CK, Makepeace BL, Njongmeta LM, Tanya VN, Bain O, et al. (2006) Eosinophils contribute to killing of adult *Onchocerca ochengi* within onchocermata following emilination of *Wolbachia*. Microbes Infect 8: 2698-2705.
287. Junker K, Bain O, Boomker J (2006) *Eustrongylides* sp. (Nematoda: Dioctophymatoidea) from the stomach of a Nile crocodile, *Crocodylus niloticus* Laurenti, 1768, in Botswana. Onderstepoort J Vet Res 73: 315-317.
288. Morimoto N, Korenaga L, Yagu K, Kagei N, Fijieda M, et al. (2006) Morphological observation and the effects of artificial digestive fluids on the survival of *Diploscapter coronata* form a japanese patient. J Helminthol 80: 341-348.
289. Lhermitte N, Bain O, Hering-Hagenbeck S (2007) Three species of *Skrjabinelazia* (Nematoda: Seuratidae) parasite of Geckonidae and Lacertidae from South Africa, Europe and Australia. Syst Parasitol 67: 125-137.
290. Uni S, Bain O, Agatsuma K, Harada, Matsubayashi M, et al. (2007) *Onchocerca eberhardi* n. sp. (Filarioidea, Nematoda) from the sika deer in Japan; relationhips between species parasitic in cervids and bovids in holarctic region. Parasite 14: 199-211.
291. Ivanova E, Spiridonov S, Bain O (2007) Ocular oxyspirurosis of primates in zoos: intermediate host, worm morphology, and probable origin of the infection in the Moscow zoo. Parasite 14: 287-298.
292. Taylor MD, Harris A, Babayan S, Bain O, Culshaw A, et al. (2007) CTLA-4 and CD4+ CD25+ regulatory T cells inhibit protective immunity to filarial parasites *in vivo*. J Immunol 179: 4626-4634.
293. Wisely SM, Howard JG, Williams SA, Bain O, Santymire RM, et al. (2008) An unidentified filarial species in wild populations of the black-footed ferret (*Mustela nigripes*). J Wildl Dis 44: 53-64.
294. Lhermitte N, Bain O, Virga A (2008) *Skrjabinelazia rizzoi* n. sp. (Nematoda: Seuratoidea) from a Sicilian lacertid, with comments on specific and biological diversity in the genus. Parasite 15: 45-52.
295. Krief S, Jamart A, Mahé S, Leendertz FH, Mätz-Rensing K, et al. (2008) Clinical and pathological manisfestation of oesophagostomosis in African great apes. Does self-medication in wild apes influence disease progression? J Med Primatol 37: 188-195.
296. Fukuda M, Takaoka H, Uni S, Bain O (2008) Infective larvae of five *Onchocerca* species from experimentally infected *Simulium* species in an area of zoonotic onchocerciasis in Japan. Parasite 15: 111-119.
297. Attout T, Martin C, Babayan SA, Kozek WJ, Bazzochi C, et al. (2008) Pleural celluar reaction to the filarial infection *Litomosoides sigmodontis* is determined by the moulting process, the worm alteration, and the host strain. Parasitol Int 57: 201-211.
298. Planade B, Bain O, Lena J, Joly P (2008) *Gyrinicola chabadamsoni* n. sp. and *G. tba* (Dinnik 1933) (Nematoda, Oxyuroidea) parasite in tadpoles of the hybridogenetic complex *Rana lessonae-esculenta* (Amphibia, Ranoidea). Zootaxa 1764: 27-39.
299. Bain O, Casiraghi M, Martin C, Uni S (2008) The Nematoda Filarioidea: critical analysis linking molecular and traditional approaches. Parasite 5: 342-348.
300. Allen JE, Adjei O, Bain O, Hoerauf A, Hoffmann WH, et al. (2008) Of mice cattle and humans: the immunology and treatment of river blindness. PLoS NTD 2:e217.
301. Ishii Y, Choochote W, Bain O, Fukuda M, Otsuka Y, et al. (2008) Seasonal and diurnal biting activities and zoonotic filarial infections of two *Simulium* species in northern Thailand. Parasite 15: 121-129.
302. Hira PR, Al-Buloushi A, Khalid N, Iqbal J, Bain O, et al. (2008) Case Report: Zoonotic filariasis in the Arabian Peninsula: autochthonous onchocerciasis and dirofilariasis. Am J Trop Med Hyg 79: 739-741.
303. Lhermitte-Vallarino N, Barbuto M, Ineich I, Wanji S, Lebreton M, et al. (2008) First report of *Rhabdias* (Nematoda: Rhabdiasoidea) from lungs of montane chameleons in Cameroon: description of two new species and notes on biology. Parasite 15: 553-564.
304. Taylor MD, Van Der Werf N, Harris A, Graham AL, Bain O, et al. (2009) Early recruitment of natural CD4+ Foxp3+ Treg cells by infective larvae determines the outcome of filarial infection. Eur J Immunol 39: 192-206.
305. Junker K, Bain O, Boomker J (2009) Helminth parasites of Natal long-fingered bats, *Miniopterus natalensis* (Chiroptera: Miniopteridae), in South Africa. Onderstepoort J Vet Res 75: 261-265.
306. Junker K, Barbuto M, Casiraghi M, Martin C, Uni S, et al. (2009) *Litomosa chiropterorum* Ortlepp, 1932 (Nematoda: Filarioidea) from a South African Miniopterid: redescription, *Wolbachia* screening and phylogenetic relationships with *Litomosoides*. Parasite 16: 43-50.
307. Ferri E, Lo N, Martin C, Barbuto M, Bain O, et al. (2009) Integrated taxonomy: traditional approach and DNA barcoding for the identification of filarioid worms and related species (Nematoda). Front Zool 6:1.
308. Lhermitte-Vallarino N, Junker K, Bain O (2009) Reappraisal of the specific status of *Rhabdias* (Nematoda) from Malagasy chameleons in the Paris Museum collection. Parasite 16: 111-123.
309. Attout T, Hoerauf A, Dénécé G, Debrah AY, Marfo-Debrekyei Y, et al. (2009) Lymphatic vascularisation and involvement of Lyve-1+ macrophages in the human *Onchocerca* nodule. PLoS One 4:e8234.
310. Lhermitte-Vallarino N, Barbuto M, Junker K, Boistel R, Ineich I, et al. (2009) *Rhabdias rhampholeonis* n. sp. and *Rhabdias mariauxi* n. sp. (Nematoda, Rhabdiasoidea), first lung worms from leaf chameleons: description, molecular evidence and notes on biology. Parasitol Int 58: 375-383.
311. Junker K, Lhermitte-Vallarino N, Barbuto M, Ineich I, Wanji S, et al. (2010) New species of *Rhabdias* (Nematoda, Rhabdiasidae) from Afrotropical anurans, including molecular evidence and notes no biology. Folia Parassitol 57: 47-61.
312. Krief S, Vermeulen B, Lafosse S, Kesenene JM, Nieguitsila A, et al. (2010) Nodular worm infection in wild chimpanzees in western Uganda: a risk for human health? PloS NTD, 4:e630.
313. Laaksonen S, Saari S, Nikander S, Oksanen A, Bain O (2010) Lymphatic dwelling filarioid nematodes in reindeer (*Rangifer tarandus tarandus*) (Cervidae) in Finland, identified as *Rumenfilaria andersoni* Lankester & Snider, 1982 (Nematoda: Onchocercidae: Splendidofilariinae). Parasite 17: 23-31.
314. Fukuda M, Otsuka Y, Uni S, Bain O, Takaoka H (2010) Genetic evidence for the presence of two species of *Onchocerca* from the wild boar in Japan. Parasite 17: 33-37.
315. Fukuda M, Otsuka Y, Uni S, Bain O, Takaoka H (2010) Molecular identification of infective larvae of three species of *Onchocerca* found in wild-caught females of Simulium bidentatum in Japan. Parasite 17: 39-45.
316. Lhermitte-Vallarino N, Barbuto M, Junker K, Boistel R, Bain O (2010) *Rhabdias* (Nematoda, Rhabdiasidae) from Chamaeleonidae (Sauria): two new species from *Trioceros ellioti* in east Africa and one from *Brookesia superciliaris* in Madagascar. Parasite 17: 91-105.
317. Lhermitte-Vallarino N, Barbuto M, Junker K, Wanji S, Ineich I, et al. (2010) The lung nematode parasites of the genus *Rhabdias* (Rhabdiasidae): diversity and biology in the Chamaeleonidae (Squamata) and hypothesis on their evolution. Bull Soc Zool France 135: 109-118.
318. Bercion R, Heaulme M, Viant E, Moravec F, Bain O (2010) [Description of a case of infection due to *Dracunculoidea* in French Polynesia.](http://www.ncbi.nlm.nih.gov/pubmed/20486362) Med Trop 70:98.
319. Michalski ML, Bain O, Fischer K, Fischer PU, Kumar S, et al. (2010) Identification and phylogenetic analysis of *Dirofilaria ursi* (Nematoda: Filarioidea) from Wisconsin black bears (Ursus americanus) and its *Wolbachia* endosymbiont. J Parasitol 96: 412-419.
320. Uni S, Boda T, Daisaku K, Ikura Y, Mauyama H, et al. (2010) Zoonotic filariasis caused by *Onchocerca dewittei japonica* in a resident of Hiroshima Prefecture, Honshu, Japan. Parasitol Int 59: 477-480
321. Pereira FB, Souza SL, Bain O. (2010) [*Oswaldofiaria chabaudi* n. sp. (Nematoda: Onchocercidae) from a South American tropidurid lizard (Squamata: Iguania) with an update on Oswaldofilariinae.](http://www.ncbi.nlm.nih.gov/pubmed/21275236) Parasite 17: 307-318.
322. Babayan S, Read AF, Lawrence RA, Allen JE (2010) Filarial parasites develop faster and reproduce earlier in response to host immune effectors which determine filarial life expectancy. PLos Biology. 19:e1000525.
323. Hansen RD, Trees AJ, Bah GS, Hetzel U, Martin C, et al. (2011) A worm's best friend: recruitment of neutrophils by *Wolbachia* confounds eosinophil degranulation against the filarial nematode *Onchocerca ochengi.* Proc Biol Sci 278: 2293-2302.
324. Furst Von Lieven A, Uni S, Ueda K, Barbuto M, Bain O (2011) *Cutidiplogaster manati* n. gen, n. sp. (Nematoda: Diplogastridae) from skin lesions of a West Indian manatee (Sirenia) from the Okinawa Churaumi Aquarium. Nematology 13: 51-59.
325. Otranto D, Sakru N, Testini G, Gürlü VP, Yakar K, et al. (2011) [Case report: First evidence of human zoonotic infection by *Onchocerca lupi* (Spirurida, Onchocercidae).](http://www.ncbi.nlm.nih.gov/pubmed/21212202) Am J Trop Med Hyg 84: 55-58.
326. Fukuda M, Otsuka Y, Uni S, Boda T, Daisaku H, et al. (2011) Zoonotic onchocerciasis in Hiroshima, Japan, and molecular analysis of a paraffin section of the agent for a reliable identification. Parasite 18: 185-188.
327. Guerrero R, Bain O (2011) Study of types of some species of *Filaria* (Nematoda) parasites of small mammals described by von Linstow and Molin. Parasite 18: 151-161.
328. Guerrero R, Bain O, Martin C, Barbuto M (2011) A new species of *Litomosides* (Nematoda : Onchocercidae), parasite of *Nectomys palmipes* (Rodentia : Cricetidae : Sigmodontinae) from Venezuela: description, molecular evidence, *Wolbachia pipientis* screening. Folia Parasitologia. 58: 149-156.
329. Specht S, Frank JK, Alferink J, Dubben B, Layland LE, et al. (2011) CCL17 controls mast cells for the defense against filarial larval entry. J Immunol 186: 4845-4852.
330. Otranto D, Diniz D. G, Dantas-Torres F, Casiraghi M, De Almeida IN, et al. (2011) [Human intraocular filariasis caused by *Dirofilaria* sp. nematode, Brazil.](http://www.ncbi.nlm.nih.gov/pubmed/21529396) Emerg Infect Dis 17: 863-866.
331. Bain O, Otranto D, Diniz DG, dos Santos JN, de Oliveira NP, et al. (2011) [Human intraocular filariasis caused by *Pelecitus* sp. nematode, Brazil.](http://www.ncbi.nlm.nih.gov/pubmed/21529397) Emerg Infect Dis 17: 867-869.
332. Otranto D, Brianti E, Dantas-Torres F, Weigl S, Latrofa MS, et al. (2011) [Morphological and molecular data on the dermal microfilariae of a species of *Cercopithifilaria* from a dog in Sicily.](http://www.ncbi.nlm.nih.gov/pubmed/21705146) Vet. Parasitol 182: 221-229.
333. Ferri E, Bain O, Barbuto M, Martin C, Lo N, et al. (2011) [New insights into the evolution of *Wolbachia* infections in filarial nematodes inferred from a large range of screened species.](http://www.ncbi.nlm.nih.gov/pubmed/21731626) PLoS One 6:e20843.
334. Guillot J, Vermeulen B, Lafosse S, Chauffour S, Cibot M, et al. (2011) Nematodes of the genus *Oesophagostomum*: an emerging risk for humans and apes in Africa? Bull Acad Natl Med 195:1955-1963
335. Brianti E, Otranto D, Dantas-Torres F, Weigl S, Latrofa MS, et al. (2012) [*Rhipicephalus sanguineus* (Ixodida, Ixodidae) as intermediate host of a canine neglected filarial species with dermal microfilariae.](http://www.ncbi.nlm.nih.gov/pubmed/21831524) Vet Parasitol 183:330-337.
336. Landmann F, Bain O, Martin C, Uni S, Taylor MJ, et al. (2012) Both asymmetric mitotic segregation and cell-to-cell invasion are required for stable germline transmission of *Wolbachia* in filarial nematodes. Biology open 1: 536-547.
337. Otranto D, Latrofa MS, Brianti E, Annoscia G, Parisi A, et al. (2012) An assessment of genetic variability in the mitochondrial cytochrome c oxidase subunit 1 gene of *Cercopithifilaria* sp. (Spirurida, Onchocercidae) from dog and *Rhipicephalus sanguineus* populations. Mol Cell Probe 26: 81-89.
338. Robles Mdel R, Bain O, Navone GT (2012) Description of A New Capillariinae (Nematoda: Trichuridae) from *Scapteromys aquaticus* (Cricetidae: Sigmodontinae) from Buenos Aires, Argentina. J Parasitol 98: 627-639.
339. Fall EH, Diagne M, Junker K, Duplantier JM, et al. (2012) Development of *Trichosomoides nasalis* (Nematoda : Trichinelloidea) in the murid host : evidence for larval growth in striated muscle fibres. Parasite. 19: 19-29.
340. Otranto D, Brianti E, Latrofa MS, Annoscia G, Weigl S, et al. (2012) On a *Cercopithifilaria* sp. transmitted by *Rhipicephalus sanguineus*: a neglected, but widespread filarioid of dogs. Parasit Vectors. Parasit Vectors 5(1):1.
341. Ivanova E, Bain O (2012) A new genus and six new species of *Ungellidae* (Rhabditida: Drilonematoidea) parasitic in earthworms from West Africa and Haiti. Nematology 14: 457-481.
342. Takaoka H, Fukuda M, Otsuka Y, Aokic, Uni S, et al. (2012) Blackfly vectors of zoonotic onchocerciasis in Japan. Med Vet Entomol 26:372-378.
343. Brianti E, Gaglio G, Napoli E, Giannetto S, Dantas-Torres F, et al. (2012) New insights into the ecology and biology of *Acanthocheilonema reconditum* (Grassi, 1889) causing canine subcutaneous filariosis. Parasitology 139: 530-536.
344. Ziewer S, Hubner MP, Dubben B, Hoffmann WH, Bain O, et al. (2012) Immunization with *L. sigmodontis* microfilariae reduces peripheral microfilaraemia after challenge infection by inhibition of filarial embryogenesis. PLoS NTD 6:e1558.
345. Bouchery T, Denece G, Attout T, Ehrhardt K, Lhermitte-Vallarino N, et al. (2012) The chemokine CXCL12 is essential for the clearance of the Filaria *Litomosoides sigmodontis* in resistant mice. PLoS ONE 7:e34971.
346. Otranto D, Dantas-Torres F, Cebeci Z, Yeniad B, Buyukbabani N, et al. (2012) Human ocular onchocerciasis: further evidence on the zoonotic role of *Onchocerca lupi*. Parasit Vectors 25(1):84.
347. Otranto D, Brianti E, Abramo F, Gaglio G, Napoli E, et al. (2012) Cutaneous distribution and localization of *Cercopithifilaria* sp. microfilariae in dogs. Vet Parasitol 190: 143-150
348. Otranto D, Dantas-Torres F, Papadopoulos E, Petrić D, Cupina AI, et al. (2012) Tracking the vector of *Onchocerca lupi* in a rural area of Greece. Emerg Infect Dis 18: 1196-1200.
349. Lefoulon E, Gavotte L, Junker K, Barbuto M, Uni S, et al. (2012) A new type F *Wolbachia* from Splendidofilariinae (Onchocercidae) supports the recent emergence of this supergroup. Int J Parasitol 42: 1025-1036.
350. Otranto D, Brianti E, Dantas-Torres F, Miró G, Latrofa MS, et al. (2012) Species diversity of dermal microfilariae of the genus *Cercopithifilaria* infesting dogs in the Mediterranean region. Parasitology 23: 1-10.
351. Fall EH, Diagne M, Martin C, Mutafchiev Y, Granjon L, et al. (2012) *Trichosomoides nasalis* (Nematoda: Trichinelloidea) in the murid host *Arvicanthis niloticu*s: migration to the epithelium of the nasal mucosa after intramuscular development. Parasite 19: 359-365.
352. Bouchery T, Ehrhardt K, Lefoulon E, Hoffmann W, Bain O, et al. (2012) Differential tissular distribution of *Litomosoides sigmodontis* microfilariae between microfilaremic and amicrofilaremic mice following experimental infection. Parasite 19: 351-358.
353. Souza Lima S, Marun B, Alves PV, Bain O (2012). *Ochoterenella esslingeri* n. sp. (Nematoda: Onchocercidae: Waltonellinae) from *Bokermannohyla luctuosa* (Anura: Hylidae) in Minas Gerais, Brazil, with notes on *Paraochoterenella* Purnomo & Bangs, 1999. Parasite 19: 341-350.
354. Junker K, Medger K, Lutermann H, Bain O (2012) *Monanema joopi* n. sp. (Nematoda, Onchocercidae) from *Acomys (Acomys) spinosissimus* Peters, 1852 (Muridae) in South Africa, with comments on the filarial genus. Parasite 19: 331-40.
355. Masi S, Chauffour S, Bain O, Todd A, Guillot J, et al. (2012) Seasonal effects on great ape health: a case study of wild chimpanzees and Western gorillas PLoS One 7(12):e49805.
356. Choumet V, Attout T, Chartier L, Khun H, Sautereau J, et al. (2012) Visualizing non infectious and infectious *Anopheles gambiae* blood feedings in naive and saliva-immunized mice. PLoS One 7(12):e50464.
357. Uni S, Bain O, Suzuki K, Agatsuma T, Harada M, et al. (2013) *Acanthocheilonema delicata* n. sp. (Nematoda: Filarioidea) from Japanese badgers (*Meles anakuma*): Description, molecular identification, and *Wolbachia* screening. Parasitol Int 62:14-23.
358. Uni S, Bain O, Fujita H, Matsubayashi M, Fukuda M, et al. (2013) Infective larvae of *Cercopithifilaria* spp. (Nematoda: Onchocercidae) from hard ticks (Ixodidae) recovered from the Japanese serow (Bovidae). Parasite 20:1.
359. Bain O, Junker K (2013) *Trichospirura aethiopica* n. sp. (Nematoda: Rhabdochonidae) from *Malacomys longipes* (Rodentia: Muridae) in Gabon, first record of the genus in the Ethiopian Realm. Parasite 20:4.
360. Robles MR, Eberhardt MA, Bain O, Beldomenico PM. (2013) Redescription of *Echinocoleus hydrochoeri* (Travassos, 1916) (Nematoda: Trichuridae) from *Hydrochoeris hydrochaeris* Linnaeus, 1766 (Rodentia: Caviidae) from Argentina. J Parasitol. In press.
